# Gating and regulation of KCNH (ERG, EAG, and ELK) channels by intracellular domains

**DOI:** 10.1080/19336950.2020.1816107

**Published:** 2020-09-12

**Authors:** Sara J. Codding, Ashley A. Johnson, Matthew C. Trudeau

**Affiliations:** Department of Physiology, University of Maryland School of Medicine, Baltimore, MD, USA

**Keywords:** hERG, EAG K channel, LQTS, PAS domain, CNBHD cyclic nucleotide-binding domain, ELK K channel, intrinsic ligand

## Abstract

The KCNH family comprises the ERG, EAG, and ELK voltage-activated, potassium-selective channels. Distinct from other K channels, KCNH channels contain unique structural domains, including a PAS (Per-Arnt-Sim) domain in the N-terminal region and a CNBHD (cyclic nucleotide-binding homology domain) in the C-terminal region. The intracellular PAS domains and CNBHDs interact directly and regulate some of the characteristic gating properties of each type of KCNH channel. The PAS-CNBHD interaction regulates slow closing (deactivation) of hERG channels, the kinetics of activation and pre-pulse dependent population of closed states (the Cole-Moore shift) in EAG channels and voltage-dependent potentiation in ELK channels. KCNH channels are all regulated by an intrinsic ligand motif in the C-terminal region which binds to the CNBHD. Here, we focus on some recent advances regarding the PAS-CNBHD interaction and the intrinsic ligand.

## Introduction

In this review, we discuss the discovery that point mutations in the intrinsic ligand disrupt the PAS-CNBHD interaction as measured with electrophysiology and FRET in hERG channels [1,2], the identification of a small molecule (chlorpromazine) that targets the PAS and disrupts its regulatory function in EAG channels [3] and a high (1.5 Angstrom) resolution X-ray crystal structure of the hERG CNBHD which reveals new hydrogen bond contacts for the intrinsic ligand and identifies a salt bridge near the intrinsic ligand that is necessary for hERG channel function [4].

## Background

### Organization of KCNH channels

**Figure 1.**
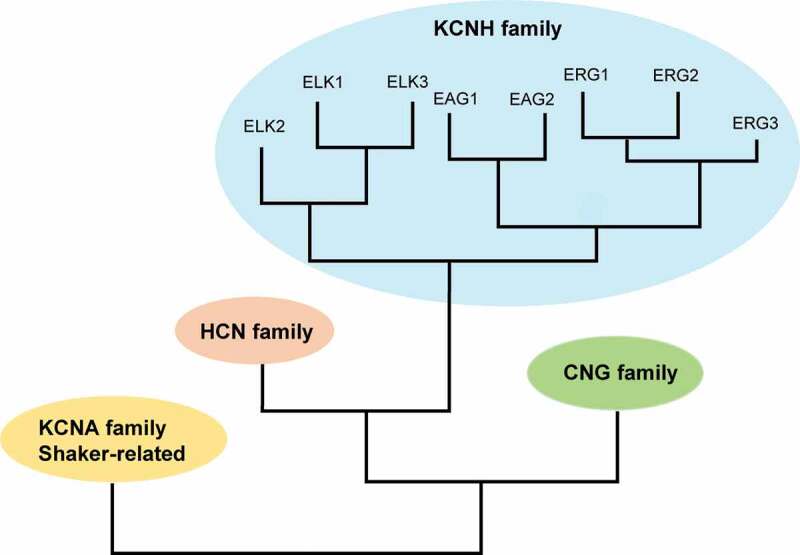
Cladogram of channels in the KCNH family. Comparison of ERG (KCNH2), EAG (KCNH1), ELK (KCNH3), CNG, HCN and Kv (KCNMA) channels.

KCNH channels comprise the ERG (ether á go-go related, KCNH2), EAG (ether á go-go, KCNH1), and ELK (ether á go-go like; KCNH3) families of voltage-activated potassium channels [5,6] and are separated into different groups based on homology (Figure 1). ERG, EAG, and ELK channels are homologous to the CNG (cyclic nucleotide-gated) and HCN (hyperpolarization-activated, cyclic nucleotide-regulated) channels [7,8]. KCNH channels are more closely related to CNG and HCN channels than to other voltage-activated (Kv, KCNMA) K channels [9].

### KCNH channel physiology and pathophysiology

#### ERG channels

**Figure 2.**
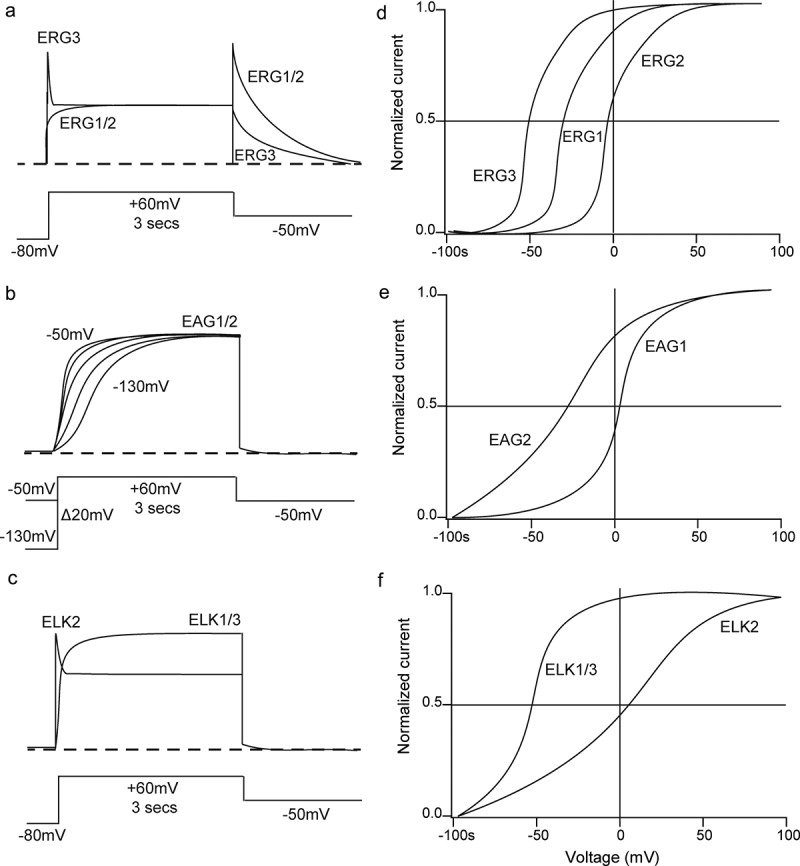
Functional characteristics of KCNH channel currents. Schematic drawings of characteristic currents from A) ERG, B) EAG and C) ELK channels. Schematic of voltage-activation relationships for D) ERG, E) EAG and F) ELK channels.

ERG1 is distinguished from other KCNHs because ERG1 is expressed in the mammalian myocardium and forms the native I_Kr_ current (rapid component of the delayed rectifier current) in cardiac myocytes [10–12]. I_Kr_ is a major repolarizing current for the late phase of the cardiac action potential (AP) [11]. ERG1 channels have slow activation, rapid inactivation, fast recovery from inactivation, and slow deactivation, giving rise to their characteristic large “tail” current with repolarization [12] (Figure 2a). ERG1a subunits (the original ERG isolate) and ERG1b subunits (a short isoform of ERG1a) form ERG1a/ERG1b heteromeric channels to encode I_Kr_ [13–17]. Genetic mutations in human ERG1a (hERG1a) or ERG1b (hERG1b) cause Type 2 Long QT syndrome which can lead to sudden death cardiac arrhythmias [18–20]. A common, acquired form of LQTS is primarily due to the inhibition of hERG1 channels (and native I_Kr_ in the heart) by drugs and pharmaceuticals [12,21,22]. The roles of ERG2 and ERG3 are less understood than that of ERG1. ERG2 and ERG3 are not expressed in the heart but ERG1a, ERG1b, ERG2, and ERG3 are widely distributed in the CNS [23–25]. ERG currents play a role in spike frequency in neurons [26], and ERG inhibitory drugs increase firing in spinal neurons [27]. ERG2 has currents similar to that of ERG1a (Figure 2a), but ERG3 has a hyperpolarization-shifted voltage–activation relationship compared to that of ERG1 and ERG2 (Figure 2d) which may account for the early inactivating current reported for ERG3 (Figure 2a) [25].

#### EAG channels

EAG1 and EAG2 are characterized by delayed rectifier K^+^ currents (Figure 2b) in heterologous expression systems [28,29]. A characteristic feature of EAG channels is slowed activation with a hyperpolarized holding potential (Figure 2b) [28], reminiscent of the Cole-Moore shift [30]. EAG2 has a voltage–activation relationship that is hyperpolarized compared to that of EAG1 (Figure 2e). EAG channels are widely distributed in the CNS [29,31]. Mice that are genetically null for EAG1 do not display gross defects in development, behavior, or in firing patterns in Purkinje cell neurons [32]. However, genetic mutations in human EAG1 are associated with the Temple-Barrister (TB) syndrome [33] and Zimmerman-Laband (ZL) syndrome [34], both of which are defined by facial and digital dysplasia, intellectual disability, and epilepsy. Mutant EAG1 channels from TB or ZL patients have gain-of-function phenotypes [33,34]. EAG1 and 2 channels are upregulated in a wide array and high proportion of cancer cell lines [35,36], and specific reduction of EAG with shRNAs [37], drugs (astemizole) [38] or antibodies [39] reduces cell growth [40].

#### ELK channels

Compared to ERG or EAG, less is known about the physiological role or native correlate of ELK channels. ELK1 and ELK3 are delayed-rectifier currents [41,42] whereas ELK2 has an early inactivating peak (Figure 2c) due to a C-type inactivation mechanism [43]. A defining characteristic of ELK channels is a voltage-dependent potentiation (VDP), in which longer depolarizing pulses and PI(4,5)P2 shifts the conductance–voltage relationship to more hyperpolarized potentials and slows down deactivation, consistent with a slow transition to a mode that favors channel opening [44]. hERG channels also exhibit VDP [45–47]. ELK channels are distributed in the CNS [41,43], including cortex and hippocampus [48]. As the voltage-activation relationship for all ELK channels expressed heterologously is hyperpolarization-shifted (Figure 2f), native ELK channels may be open at voltages near E_K_ in native cells and contribute to control of subthreshold activity in neurons. This idea is supported in studies of mice null for ELK2 channels (ELK2 ^−/-^). Hippocampal CA1 pyramidal neurons from ELK2 ^−/-^ mice have an increase in firing rate, more positive resting membrane potential, and more negative threshold to AP firing as compared to control CA1 neurons. Similarly, a specific inhibitor (CX4) of ELK2 channels makes V_m_ more positive, reduces the K^+^-selective current and increases AP firing in CA1 neurons from wild-type mice, but not in CA1 neurons from ELK2 ^−/-^ mice. Furthermore, ELK2 ^−/-^ mice have a higher incidence of PTZ-induced tonic-clonic seizures than control mice (Zhang et al., 2010). The ELK2 ^−/-^ hyperexcitability phenotype is consistent with ELK2 channels acting as a subthreshold K^+^ current in CA1 neurons [49,50].

### KCNH subunit structural organization

**Figure 3.**
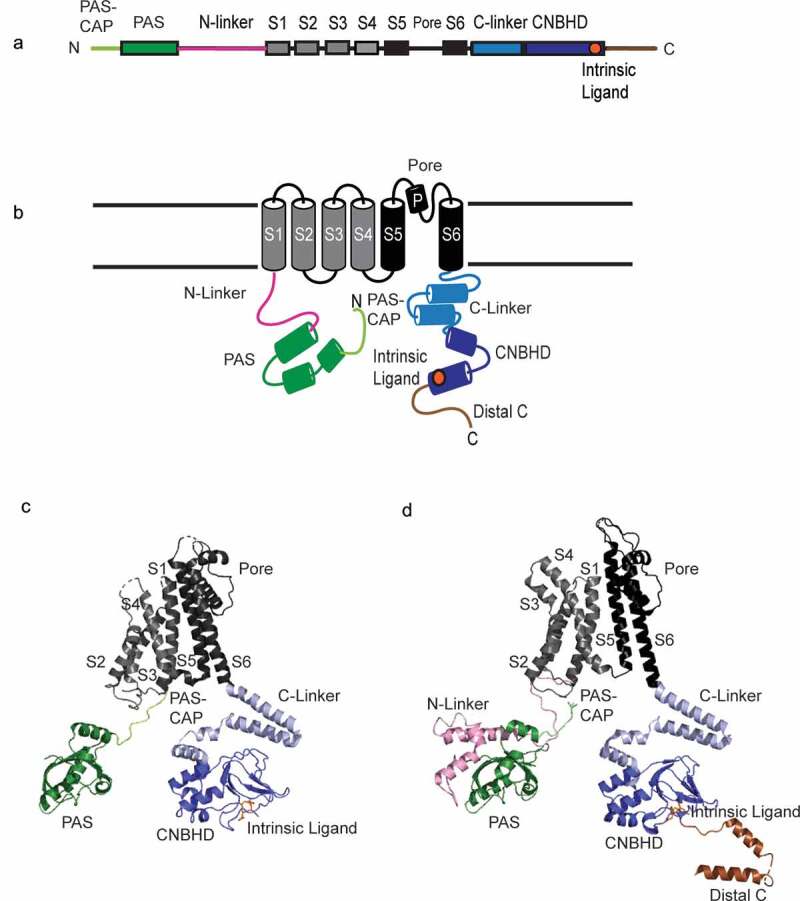
KCNH subunits. (a) Linear scheme of KCNH channel subunit, (b) Topology of KCNH channel subunit (c) Ribbon diagram of single hERG subunit (PDB 5VA2) and (d) ribbon diagram of single EAG channel subunit (PDB 5K7L). PAS-CAP = light green, PAS = green, N-linker = pink, Voltage sensing domain (VSD) = gray, Pore domain = black, C-linker = light blue, CNBHD = blue, Intrinsic ligand = orange, distal C-terminal domain = brown.

KCNH channel subunits have six transmembrane domains comprising a voltage-sensor domain (VSD) formed from the S1-S4 transmembrane domains and a Pore domain formed from the S5-Pore loop-S6 domains (Figure 3). KCNH subunits contain an intracellular N-terminal region that contains a Per-Arnt-Sim (PAS) domain, a PAS-CAP, and an N-linker domain (Figure 3a,b). The PAS and PAS-CAP together are also known as the “eag domain” since eag domains are a shared feature of KCNH (eag-family) channels. But here, for clarity, we will refer to the eag domain as the PAS domain as suggested previously [51]. All KCNH channels have an intracellular C-terminal region that contains a C-linker domain and cyclic nucleotide-binding homology domain (CNBHD). An intrinsic ligand motif, characterized by a beta strand with a conserved, three amino acid sequence (FNL in hERG and YNL in ELK and EAG), is located at the distal end of the CNBHD [52,53]. A C-terminal region is located distal to the CNBHD. Three-dimensional structures of a single hERG channel subunit (Figure 3c) [54] and a single rat EAG (rEAG) channel subunit (Figure 3d) [55] confirm the structural organization of the domains within KCNH channels. The transmembrane VSD and Pore domain of a single subunit are located nearby each other in the plasma membrane. The organization of the VSD shows the S4 helix surrounded by the S1, S2, and S3 domains and tilted away from the S5 domain and the rest of the Pore domain. The S4-S5 linker domain that connects the VSD to the Pore domain is noticeably short (approximately four amino acids) in KCNH channels relative to that of other Kv channels (approximately 14 amino acids). The intracellular PAS-CAP is pointed up, with its N-terminus positioned near the transmembrane domains. The PAS domain is located below the VSD. The C-linker and CNBHDs are intracellular and distant from the PAS domain from the same subunit. For hERG, the role of the N-linker is not well understood and is not included in structural studies. In hERG channels, the distal C-terminal region binds to the regulatory protein TRIOBP-1 [56], but this region was not included in structural studies [54]. In rEAG a novel N-linker structure is positioned adjacent to the PAS and the distal C-terminal region has two alpha helices and is connected to the CNBHD via a linker region. Both the N-linker and distal C-terminal regions bind to calcium-calmodulin [55].

### Features of KCNH channel tetramers

**Figure 4.**
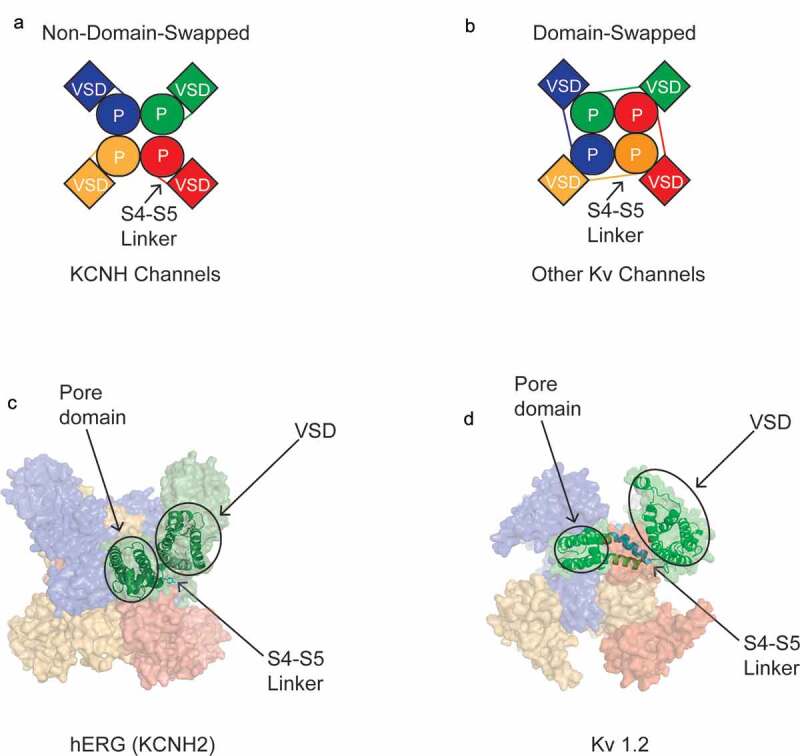
Non-domain-swapped VSD and Pore Domains in KCNH tetramers. Schematic of top-down view of (a) a representative KCNH tetramer and (b) a representative Kv channel tetramer. Top-down view of (c) hERG tetramer CryoEM structure and (d) Kv1.2 tetramer X-ray crystallography structure (PDB 3LUT). Each individual subunit in A-D is a different color to highlight non-domain swapping versus domain-swapping. Ribbon diagram of one channel subunit in C and D overlays the space-filling depiction of one subunit of the tetramer. The S4-S5 linker in C,D is depicted as cyan.

hERG and rEAG channel tetramers have a novel arrangement of functional domains compared to that of other voltage-activated K channels (Figure 4). KCNH channels have a non-domain-swapped configuration between the VSD and Pore [54,55], in which the VSD is adjacent to the Pore domain in the same subunit (Figure 4a,c). Non-domain-swapped VSD and Pore domain arrangements are also found in CNG and HCN channels [57–59]. In contrast, other voltage – activated K channels have a domain-swapped arrangement where the VSD of one subunit is positioned near the Pore domain of an adjacent subunit, as in KCNQ1 channels [60] and as shown for Kv1.2 (Figure 4b,d) [61–63].

Non-domain-swapped VSDs and Pore domains in hERG and rEAG are accompanied by a short S4-S5 linker domain (Figure 4a,c) [54,55]. In comparison, Kv1.2 channels have a longer S4-S5 linker (Figure 4b,d) [61–63]. The short S4-S5 linker in KCNH has led to the hypothesis that it may not act as a mechanical lever between the S4 and S5 to open the activation gate in KCNH channels, unlike the role of the S4-S5 linker in Kv1.2 channels, and instead the coupling of VSD movement to pore opening in KCNH channels may be fundamentally different than in Kv channels [54,55]. A non-mechanical role for the KCNH S4-S5 linkers is consistent with studies in which the ERG or EAG channel S4-S5 linker is cut but channels maintain voltage-dependent activation [64,65].

**Figure 5.**
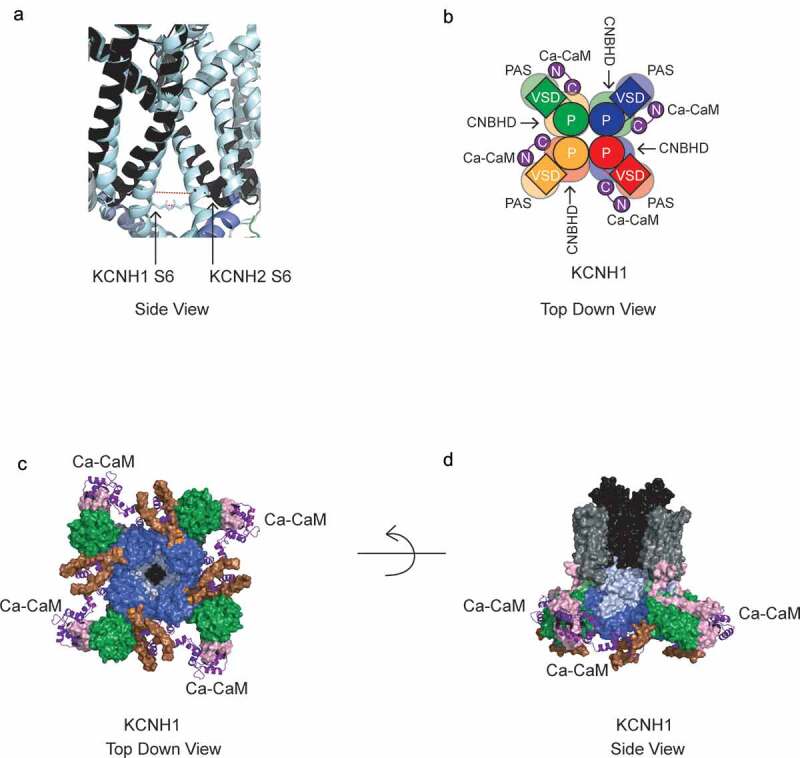
KCNH activation gates and Ca-CaM association with KCNH1. (a) Side view of overlay of closed EAG activation gate (light blue) and open hERG activation gate (black). Dashed lines depict distances between the Q476 residues in the lower S6 domain in EAG, which result in a pore radius of less than 1 angstrom and the Q664 residues in the lower S6 of hERG, which result in a pore radius of approximately 5 angstroms. (b) Schematic of top-down view of KCNH1 channels associated with four Ca-CaM proteins (purple) indicating intersubunit interaction with the channel. (c) Bottom-up view of KCNH1 and Ca-CaM (purple) indicating that the N lobe binds the N-linker domain (pink) distal to the PAS domain (green) and the C lobe binds the C-terminal region (brown) distal to the CNBHD (blue) from an adjacent subunit. The transmembrane domains and most of the C-linker were removed for clarity. (d) Side view of KCNH1 with three (of four) Ca-CaMs. Same colors as in C plus the addition of the C- linker (light blue), VSD (gray) and Pore domain (black).

A notable difference between the hERG and rEAG structures is that while the S4 voltage sensors were both in a similar activated conformation (Figure 3c,d), the S6 activation gate in hERG is open, whereas the S6 activation gate in EAG is closed (Figure 5a). The likely reason for capturing the closed structure of the activation gate is that rEAG is bound to calcium-calmodulin (Ca-CaM) (Figure 5b-d) and Ca-CaM inhibits rEAG channel currents. The Ca-CaM N-lobe is associated with the N-linker region distal to the PAS domain and the C-lobe density is associated with the C-terminal region distal to the CNBHD of the adjacent subunit (Figure 5b-d) [55,66]. In contrast, hERG is not associated with Ca-CaM [54].

### Regulation of KCNH gating by intracellular domains

#### PAS domains

All KCNH channels (except for the ERG1b isoform) have a PAS domain in the N-terminal region (Figure 3). Structures of PAS domains in isolation [67–72], in the context of (nearly) full-length hERG and full-length EAG channels [54,55], or co-crystallized with the CNBHD from EAG channels [73], all show that PAS has a central beta sheet flanked by alpha helices (Figure 3c,d). PAS domains regulate the characteristic slow deactivation gating in hERG1a (Figure 2a). Deletion of the PAS markedly accelerates (by at least fivefold) deactivation in hERG [67,74–76]. An intriguing feature of the hERG PAS domain is that it can be re-applied *in trans* as a separate piece to hERG channels with an engineered deletion of the PAS domain to recapitulate slow deactivation [67,77–82], meaning that the PAS does not require a peptide bond to the rest of the channel for its regulatory function (see Figure 8). In EAG channels, the EAG PAS regulates the voltage dependence of activation and is necessary for the dependence of activation on the pre-pulse voltage (similar to the Cole-Moore shift; Figure 2b) [83,84]. In ELK channels, the ELK PAS domain regulates deactivation and voltage-dependent potentiation (VDP) [44]. Thus, the PAS domain regulates gating to produce some of the defining features of each KCNH channel type.

#### The PAS-CAP

The PAS-CAP refers to the amino acids at the N-terminal end proximal to the PAS domain in KCNH channels (Figure 3). The PAS-CAP has a coil adjacent to the PAS and an extended structure [68,70,71] that points the amino terminus upward toward the S4, S4-S5 linker, and C-linker in hERG and rEAG channels in CryoEM structures (Figure 3c,d) [54,55]. The PAS-CAP regulates deactivation gating in hERG channels [70,76], the Cole-Moore shift, and activation time course in EAG channels [83,84] and VDP and dynamics of the intrinsic ligand in ELK channels [85]. In hERG, the deletion of the PAS-CAP has a similar effect on deactivation gating as deletion of the entire PAS domain [67,76]. But, the PAS-CAP deletion does not alter inactivation gating, unlike deletion of the entire PAS, which also slows hERG inactivation [76]. A recent investigation comparing the PAS-CAP in structures of the rEAG and hERG channel shows that deletion of residues 3–13 of the PAS-CAP rendered rEAG channels insensitive to regulation by Ca-CaM [84] whereas another study reported a potentiation by Ca-CaM by deletion of the PAS-CAP [86]. The PAS-CAP can be re-applied to hERG channels that lack the PAS-CAP to regulate hERG channel deactivation, showing that the PAS-CAP makes a direct interaction with the rest of the hERG channel [75].

#### C-linker and CNBHD

The C-linker of KCNH channels is located distal to the S6 domain and is followed by the CNBHD (Figure 3). Both domains are homologous to the C-linker and CNBD (cyclic nucleotide-binding domain) of HCN and CNG channels [7,8]. Structures of the C-linker and CNBD from HCN2 channels [87] and the C-linker and CNBHD of ELK or ERG [52,53] are broadly similar, including four helices in the C-linker and two helices, a central beta roll, and a distal helix that compose the CNBHD (Figure 3c, d). Unlike HCN and CNG channels, KCNH channels are not directly regulated or gated by cyclic nucleotides [28,88,89]. Instead, KCNH channels are self-liganded by an intrinsic ligand [52], which we discuss below in detail (see Figure 7). The hERG CNBHD is necessary for the slow deactivation mechanism in hERG channels, as hERG1a channels with a deleted CNBHD have fast deactivation [77,80] similar to hERG channels with deletion of the PAS domain. Deletion of the hERG CNBHD also speeds up hERG activation [81]. The CNBHD is necessary for the Cole-Moore shift in EAG channels [84,90].

### PAS-CNBHD interaction

**Figure 6.**
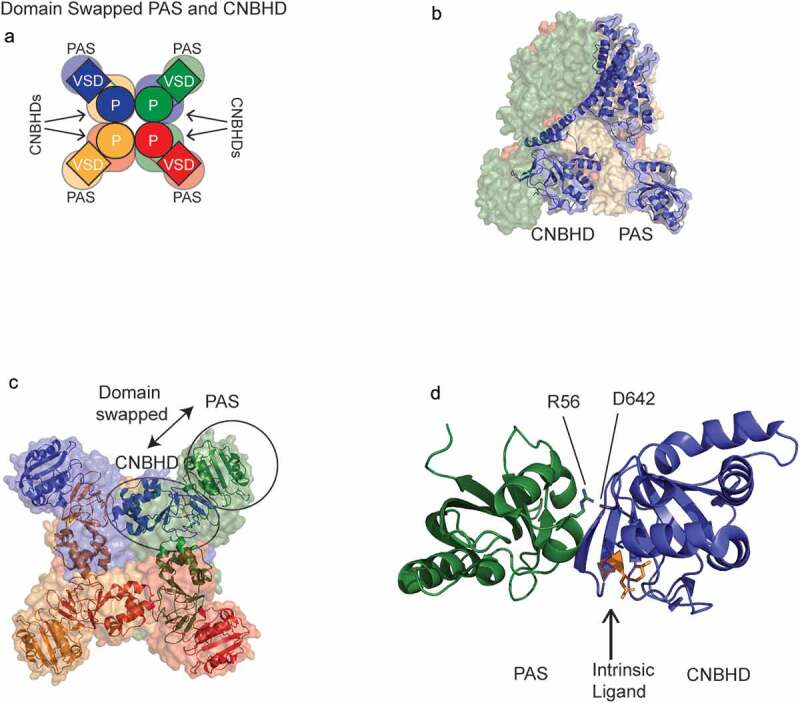
Direct PAS and CNBHD interaction in KCNH channels. (a) Top-down schematic of KCNH tetramer showing intersubunit PAS-CNBHD interaction. Single subunits are depicted as the same color. (b) Side view of hERG tetramer with one subunit shown as a ribbon to indicate that the PAS is located below the VSD and Pore of the same subunit and the CNBHD not near the PAS of the same subunit. (c) Top-down view of hERG tetramer indicating that a PAS domain from one subunit (i.e. encircled for the green subunit) interacts with the CNBHD of an adjacent subunit (i.e. encircled for the blue subunit). The transmembrane domains were removed for clarity. (d) X-ray crystallography structure of the PAS (green) and CNBHD (blue) of EAG channels in isolation from the transmembrane domains (PDB 4LLO). Intrinsic ligand (orange) in the CNBHD. Side chains R57 (green sticks) in the PAS domain and D642 (blue sticks) in the CNBHD form a salt bridge.

The PAS domain interacts directly with the CNBHD in KCNH channels (Figure 6). In hERG, the PAS-CNBHD interaction is measured in biochemical and FRET interaction assays and is necessary to regulate slow deactivation gating in hERG channels [77,80]. The PAS-CNBHD interaction is inferred to be an intersubunit interaction (Figure 6a) from functional studies because co-expression of hERG subunits with a deletion of the PAS domain and hERG subunits with a deletion of the CNBHD have partial slow deactivation gating, as if the PAS and CNBHD from the neighboring subunits made an intersubunit interaction [77,80]. A single hERG channel subunit shows that the PAS and CNBHD do not interact within the same subunit (Figure 3c,d; Figure 6b), instead a hERG tetramer shows an intersubunit interaction between the PAS and CNBHD (Figure 6c). Intersubunit, domain-swapped PAS-CNBHD interactions in EAG are found in the CryoEM structure of the EAG tetramer [55] and are likely a characteristic of KCNH channels. Thus, while the VSD and Pore have a non-domain-swapped arrangement in KCNH channels, the PAS and CNBHD interaction is domain-swapped, with the CNBHD of one subunit positioned underneath the adjacent subunit (Figure 6a-c).

The first structural data to show the PAS-CNBHD interaction in KCNH channels is an isolated, co-crystal structure from EAG channels (Figure 6d) [73]. The EAG co-crystal structure of the PAS-CNBHD reveals a large buried surface area at the interface and a functional salt bridge between R57 in the PAS and D642 in the CNBHD, showing that the interaction is physiologically relevant [73]. Regulatory salt bridges at the equivalent PAS and CNBHD residues are reported in hERG channels (R56-D803) [70] and ELK channels (R57-D861) [44].

For ELK channels there is not yet a structure at this writing. Investigations using FRET spectroscopy studies with labels placed in the ELK PAS domain and ELK CNBHD show that the PAS and CNBHD are in very close proximity (less than 15 Angstroms), as anticipated, based on the homology of ELK to EAG and hERG [44,85].

### The intrinsic ligand

**Figure 7.**
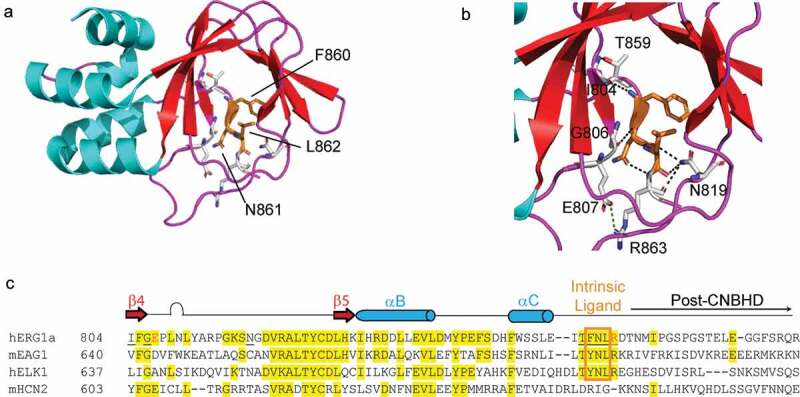
The intrinsic ligand of hERG channels (a) High-resolution X-ray crystal structure of the hERG CNBHD (PDB 6SYG) colored by secondary structure (red = β sheet, cyan = α-helix, magenta = loop). Three amino acids (F860, N861 and L862) form the core of the intrinsic ligand (orange). (b) The intrinsic ligand (orange) sits within a meshwork of interacting residues, labeled by number, and colored by atom, C = gray, N = blue, O = red, black dashed lines = H-bond. A salt-bridge (green-dashed line) between E807 and R863. (c) Alignment of C-terminal region of KCNH and HCN channels, underline = h bond network, red text = salt bridge, yellow highlight = conserved with respect to KCNH2, gold box = intrinsic ligand, secondary structure indicated by red arrow (β-sheets 4 and 5) and blue cylinder (α-helixes B and C).

Structures of the CNBHD from zebrafish ELK [52] and mosquito (ag) ERG [53] reveal that in place of a cAMP molecule bound to the CNBD as in HCN channels [87], that ELK and ERG CNBHDs have an intrinsic ligand, formed by a few amino acids at the end of the CNBHD, that sit in a binding pocket within the CNBHD (Figure 7a,b). In hERG, the amino acids F860, N861, and L862 encode a major part of the intrinsic ligand (Figure 7a). The intrinsic ligand is conserved among the KCNH channels (Figure 7c) and is a new defining feature of the KCNH family. Identification of the intrinsic ligand provided one explanation for why KCNH channels were not regulated directly by cyclic nucleotides in electrophysiology experiments [28,88,89]: KCNH channels are bound by the intrinsic ligand. Mutagenesis of the F and L residues of the FNL motif in hERG disrupts and speeds up channel deactivation [53]. Mutations in the homologous YNL motif to GNG in mouse EAG channels disrupts the Cole-Moore shift that is characteristic of EAG channels [90]. In ELK channels, mutations in the YNL motif produce a rightward shift in the voltage-activation curve [52,85]. A short peptide encoding the EAG intrinsic ligand regulates ELK channels that have a deletion of the intrinsic ligand, indicating that an exogenous intrinsic ligand can bind directly to ELK channels [85].

Recently, Haitin and colleagues solved the X-ray crystal structure of the hERG CNBHD at high (1.5 Angstrom) resolution [4]. New details provided by this structure are 1) a new, very detailed picture showing a meshwork of hydrogen bonds associated with the intrinsic ligand, including I804-F860, G806-L862, N819-L862, T859-I804, R863-N861, N819-N861, and N819-R863 (black dashed lines, Figure 7b; underlined residues, Figure 7c) and 2) the identification of a new salt bridge (green-dashed line, Figure 7b) between a residue (E807) in the CNBHD and a residue (R863) adjacent to the intrinsic ligand (red font, Figure 7c). Electrophysiology experiments show a functional role for the salt bridge in hERG channel gating and sequence alignments (Figure 7c) show the conservation of the salt bridge in other KCNH channels [4].

The intrinsic ligand is also mutated in disease in hERG. Mutations at the N in the FNL motif of hERG are associated with LQTS and lead to a lack of measurable currents at the plasma membrane and lack of the mature, N-glycosylated form of the protein [53].

Flavonoids, common dietary compounds, bind to the intrinsic ligand site in the CNBHD in EAG channels and regulate EAG channel gating (see Figure 9). Thus, the intrinsic ligand is a site that can be targeted by small molecules to modify gating [91] indicating that it may be a druggable site in KCNH channels.

The intrinsic ligand is dynamic, as determined in ELK channels using powerful dual electrophysiology and optical measurements [85]. A fluorescent amino acid genetically encoded at the YNL motif (see Figure 7c) at the N position (the FRET donor) has energy transfer with a cobalt ion bound to an engineered di-histidine motif (the FRET acceptor) located in the CNBHD. The change in transition metal (tm) FRET tracked with the voltage-dependent potentiation in ELK channels indicating that the ELK intrinsic ligand moves with voltage [85]. Whether a similarly fine movement of the intrinsic ligand occurs in hERG or EAG is not yet known.

**Figure 8.**
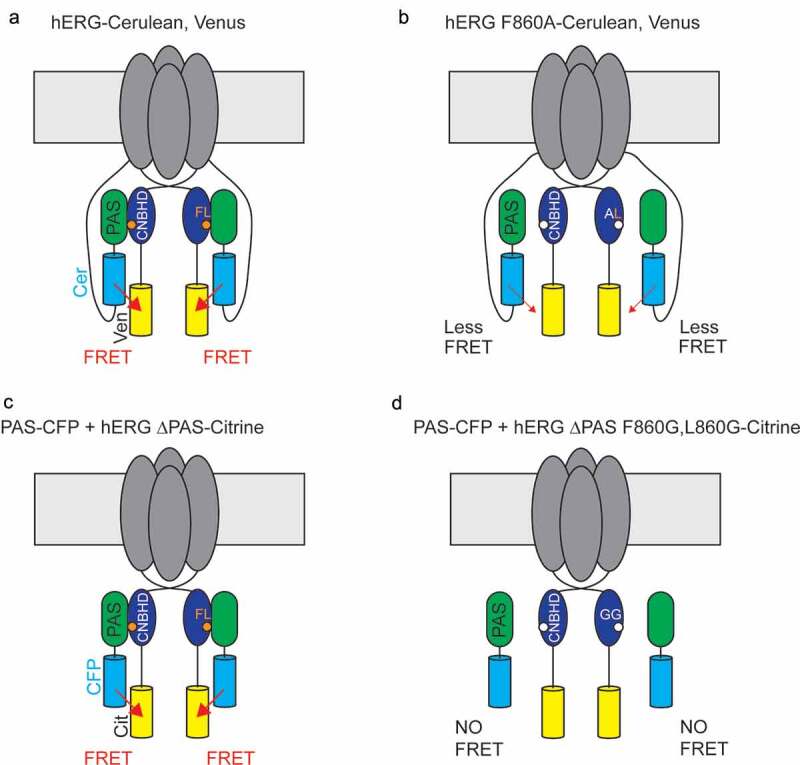
The intrinsic ligand is necessary for the PAS-CNBHD interaction. Scheme of (a) hERG FRET sensor with *in line* donor (Cerulean) and acceptor (Venus) fluorophores and robust FRET (red arrow), (b) mutation at F860 in the intrinsic ligand reduces FRET (smaller red arrow), (c) hERG FRET sensor with *in trans* donor (CFP) and acceptor (Citrine) fluorophores in which the PAS-CFP and hERG delta PAS-Citrine channel are co-expressed *in trans*. Robust FRET was detected (red arrow) (d) mutation of F860 to G and L862 to G abolishes FRET.

Recently, the intrinsic ligand was determined to be critical for the PAS-CNBHD interaction in two complementary studies (Figure 8) [1,2]. In one study, a hERG channel with a Cerulean fluorescent protein inserted *in-line* distal to the PAS and a Venus fluorescent protein inserted distal to the CNBHD had robust FRET (Figure 8a). Mutations at F in the FNL motif reduced FRET (Figure 8b) and accelerated deactivation gating [2]. In a second study, a hERG PAS domain fused to cyan fluorescent protein (CFP) was expressed *in trans* with hERG channels lacking the PAS domain and fused to Citrine fluorescent protein at the C-terminus and this configuration produced robust FRET (Figure 8c). Channels with mutations at the FNL motif abolished (F,L to G,G or L to A) or reduced (F to A) FRET (Figure 8d) and concomitantly completely or partially disrupted regulation of deactivation as measured with electrophysiology [1]. Together these results suggest that the intrinsic ligand mutations disrupt the PAS-CNBHD interaction and that the intact FNL motif is necessary for the PAS-CNBHD interaction. The FNL could either be part of a direct interaction of the CNBHD with the PAS or could allosterically influence the interaction of the CNBHD with the PAS. Future experiments will be needed to parse these different hypotheses.

### The PAS-CNBHD interaction is regulated

**Figure 9.**
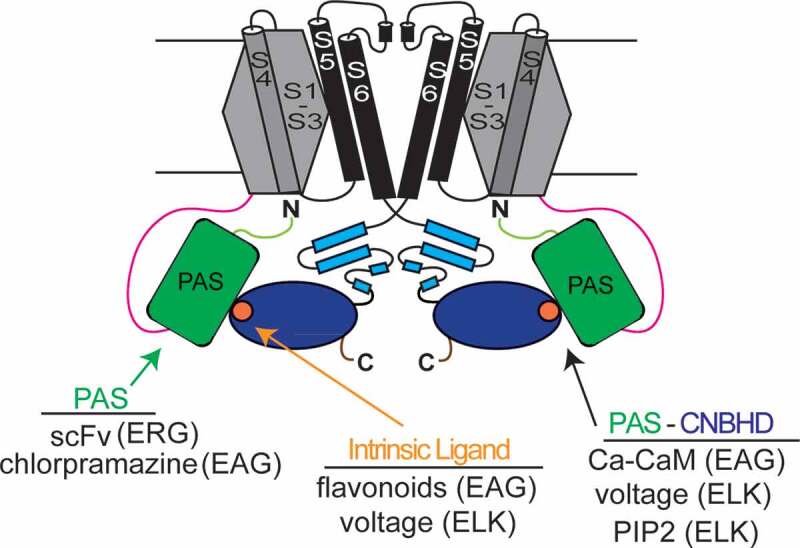
Summary of PAS-CNBHD interactions and regulators in KCNH channels. Scheme of 2 (of 4) subunits of a KCNH channel. Major domains are the VSDs (gray), Pore domains (black), PAS-CAP (light green), N-linker (pink), C-linker (light blue) and distal C-terminal region (brown). Intersubunit PAS (green) and CNBHD (dark blue) interaction is depicted with the intrinsic ligand (orange) at the surface of the CNBHD. ScFV fragments target the hERG PAS domain and impair gating and chlorpromazine binds to the EAG channel PAS and regulates gating. Flavonoids and voltage regulate the intrinsic ligand. Calcium-Calmodulin targets sites close to the PAS and CNBHD in EAG channels and inhibits channel function, and voltage and PIP2 regulate ELK channel PAS-CNBHD interactions.

The PAS-CNBHD interaction is dynamically regulated by membrane voltage. ELK channels have a voltage-dependent change in the PAS-CNBHD interaction as measured with tmFRET spectroscopy [44]. In a tmFRET experiment, a non-canonical fluorescent amino acid in the PAS domain was used as an energy donor and a transition metal ion bound to a di-histidine motif in the CNBHD was used as an energy acceptor, and changes in tmFRET with voltage steps and with PIP2 were reported, indicating that the PAS rearranges relative to the CNBHD during voltage-dependent gating in ELK channels (Figure 9).

EAG channels are strongly inhibited by calcium-calmodulin in the physiological range of intracellular calcium [92]. Ca-CaM binding sites were initially identified adjacent to the PAS domain and CNBHD using biochemical interaction assays of channel fragments and FRET interaction assays in intact channels [92–94]. Structural biology shows that the N-lobe of Ca-CaM is associated with the site adjacent to PAS domain and the C-lobe is associated with the sites adjacent to the CNBHD (Figure 5b-d) [55]. As the EAG channel structure has a closed activation gate (Figure 5a) and Ca-CaM is inhibitory, it is proposed that Ca-CaM may pull down on the CNBHD and the C-linker thus closing the EAG channel pore [55]. Interestingly, Ca-CaM inhibition of EAG requires both the PAS and CNBHD domains, as EAG channels with deletion of either the PAS or CNBHD (or both) were not inhibited by Ca-CaM [84,86] suggesting that the inhibitory action might involve regulation of the PAS-CNBHD interface (Figure 9).

In hERG channels, engineered antibodies (single-chain variable fragments; scFvs) bind the hERG PAS domain and regulate hERG channel deactivation gating [95]. The scFvs were identified in a phage display screen of PAS domains and bound to the PAS in biochemical experiments. The scFvs regulate and speed up the deactivation gating of hERG channels in electrophysiology experiments, consistent with the scFvs binding to the PAS and disrupting its regulatory function (Figure 9).

Recently, a small molecule that binds to the PAS domain of EAG channels (Figure 9) was identified [3]. Using PAS domains from KCNH channels as bait, a library of small molecules was screened in an SPR (surface plasmon resonance) assay. One compound, the antipsychotic drug chlorpromazine, bound selectively to the EAG PAS domain in the SPR assay and inhibited EAG channels in a two-electrode voltage-clamp study. EAG channels with an engineered deletion of the PAS domain were not inhibited by chlorpromazine. The mechanism for chlorpromazine inhibition may be allosteric or may involve dysregulation of the PAS-CNBHD interaction. The finding that a small molecule targeted the PAS to regulate gating is a critical advance for the KCNH field.

## Summary of recent advances

In summary, recent reports have increased our understanding of KCNH channel regulation by the intracellular PAS and CNBHDs (Figure 9). These include the identification of a small molecule, chlorpromazine, that binds to the EAG channel PAS domain and inhibits EAG channel gating [3], the high-resolution structure of the hERG CNBHD, which identifies new hydrogen bond contacts for the intrinsic ligand within the CNBHD and identifies a new salt bridge adjacent to the intrinsic ligand that is common in all KCNH channels [4] and the finding that mutations in the intrinsic ligand disrupt the PAS-CNBHD interaction in hERG channels [1,2]
